# The Complex Association between Sleep Quality, Psychological Wellbeing, and Neurodevelopmental Disorders in Childhood

**DOI:** 10.3390/jcm12103417

**Published:** 2023-05-11

**Authors:** Michele Roccella, Luigi Vetri, Marco Carotenuto, Carola Costanza

**Affiliations:** 1 Department of Psychology, Educational Science and Human Movement, University of Palermo, 90128 Palermo, Italy; michele.roccella@unipa.it; 2 Oasi Research Institute-IRCCS, Via Conte Ruggero 73, 94018 Troina, Italy; 3 Clinic of Child and Adolescent Neuropsychiatry, Department of Mental Health, Physical and Preventive Medicine, University of Campania “Luigi Vanvitelli”, 81100 Caserta, Italy; marco.carotenuto@unicampania.it; 4 Department of Sciences for Health Promotion and Mother and Child Care “G. D’Alessandro”, University of Palermo, 90128 Palermo, Italy; dottoressacostanza@gmail.com

During child development, the psychophysiological state is influenced by factors such as family routine, school experiences, stressful life events, or, in general, the environmental context in which the child grows up. Recently, the results of several studies have shown that factors mainly related to lifestyle, such as sleep quality or nutrition, can strongly influence children’s psychological well-being.

Epidemiological studies suggest that up to 25% of children under five suffer from sleep disorders, while after six years of age, this percentage progressively decreases to 10–12%. The most frequent sleep disorders are insomnia (20–30%), parasomnia or NREM sleep arousal disorders (25%), circadian rhythm sleep–wake disorders (7%), breathing-related sleep disorders (2–3%), sleep-related movement disorders (1–2%), and hypersomnolence disorders (0.01–0.20%) [1].

Sleep disorders are caused by multiple factors such as genetics, maternal diseases such as depression, attachment disorders, bad sleep hygiene, bad eating habits, and family-related problems.

Sleep problems have a significant impact on children’s daily life and well-being. They can provoke daytime sleepiness, inattention, decreased memory, poor impulse control, behavioral problems, poor academic performance and learning problems, obesity, diabetes, an increased risk of developing ADHD, oppositional defiant disorders, depressive disorders, and during adolescence, substance abuse, depression, and suicidal ideation [2].

In their research study on preschool children, Manti et al. showed that sleep-related problems play an essential role in the self-regulation of emotional skills and, consequently, may interfere with behavioral expression. Sleep disorders in young children can lead to anxiety or behavioral problems and they should be considered as a “red flag” for further assessment of psychiatric disorders [3].

Sleep problems are more frequent in children with neurodevelopmental disorders [4]. A recent systematic review by Petruzzelli et al. investigated literature data on polysomnography, sleep electroencephalography, and sleep-related questionnaires in children with autism spectrum disorders. The main objective sleep parameters in patients with autism spectrum disorders were a shorter total sleep time, higher sleep latency, and a higher number of nocturnal awakenings. These macrostructural sleep anomalies are in line with the subjective parental perception of their children’s sleep quality in terms of greater difficulties with initiating and maintaining sleep than typically developed children [5].

Sleep disorders are a significant health problem, not only among children but also among adolescents and young adults. In a three-wave prospective study, Liu et al. examined the associations between insomnia, daytime sleepiness, and depressive symptoms and found that they were widespread in and highly associated with adolescents. They assumed that teenagers are vulnerable to sleep problems due to the interaction between pubertal development, increased social or academic demands, and a likely increased risk of behavioral and emotional problems. The study found that insomnia was the most critical factor which determines daytime sleepiness and depression, leading to a negative impact on adolescents’ quality of life and an increased risk of substance use, accidents, or suicidal behavior [6].

In the same field of investigation, Babicki et al., in a cross-sectional review among Polish students conducted in 2016–2021, investigated the associations between sleep, quality of life, and drug use. They found that insufficient sleep can be associated with psychoactive substance abuse and a lower quality of life. Female and first-year college students tend to suffer more frequently from sleep disorders, and they commonly have memory, concentration, and attention problems. Therefore, the impact of sleep quality, especially among adolescents, affects their physical health and everyday functioning [7].

Children’s sleep regulation can also be determined by organic disorders, such as the alteration of the serotoninergic system involved in nociception. This would explain the frequent comorbidity between sleep perturbations and mood and anxiety disorders. Sleep disorders in childhood are also associated with the onset of migraine, and they share common pathogenetic processes involving common cerebral structures and signaling pathways [8]. Improving sleep quality could help reduce migraine-related intensity and susceptibility and vice versa. Onofri et al. in their review of the existing literature on the diagnostic assessment of the comorbidity between primary headache and sleep disorders underline the lack of data properly assessing both headache and sleep disorders. The paucity of specific diagnostic tools often underestimates this comorbidity that remains undertreated [9]. 

Similarly, Voci et al. found that sleep disturbances such as sleep onset delay, sleep duration, and night wakings are often related to migraine in the pediatric population, especially in patients with higher headache severity and lower response to acute therapy. The authors suggest that the behavioral or pharmacological treatment of one disorder can positively affect the other and vice versa [10].

Blumer et al., focusing on sleep-related breathing disorders, described some red flags that can be utilized to assess the presence of several disturbances, from frequent snoring to obstructive sleep apnea syndrome (OSAS), to prevent such disorders and improve sleep hygiene. Child caregivers should be more aware of the possible presence of sleep-related disturbance. All professionals involved in childcare should actively inquire about disturbed sleep, snoring, or mouth breathing among their young patients [11].

In particular, as Oros et al. report in their study, the early detection and treatment of OSAS is a priority for children with complex genetic conditions. The technology involved in therapy, such as non-invasive ventilation or continuous positive airway pressure and otorhinolaryngological surgery, is fundamental and the main part of a multidisciplinary approach [12].

During childhood, sleep disorders are more frequent and have significant impacts both on the child’s health and on his/her family’s health. The role of parents in the genesis and maintenance of these disorders is usually important; therefore, it is necessary to teach them the basics of children’s sleep. In children, sleep is an evolving process; thus, in the management of falling asleep and awakening, parents could accidentally determine bad associations between normal sleep and the child’s dysfunctional behavior in relation to sleep.

Sleep disorders can have significant consequences on physical and psychological health and can provoke the onset of daytime behavior disorders, mood disorders, excessive daytime weakness and concentration deficits, memory and learning disorders, growth deficits because of a significant reduction in growth hormones, endocrinological and metabolic alterations such as obesity, and a global worsening of the child’s physical and psychological health. Evidence shows that this situation often leads to increased family stress because of the parents’ own sleep deprivation. 

The negative impact of a sleep disorder on the psychophysical well-being of a child, especially if there is a coexisting neurodevelopmental disorder present, requires in more complicated cases a multispecialty, comprehensive program dedicated to the diagnosis and treatment of sleep disorders including pediatrics, internal medicine, family medicine, neurology, pulmonary medicine, psychology, psychiatry, and otolaryngology in order to define a treatment plan, including pharmacological and non-pharmacological interventions, that will be designed specifically for the patient.

Such measures will help to re-establish a physiological sleep–wake rhythm which often means creating fertile ground for the formation of the child’s psychophysical well-being. 

The literature evidence suggests that sleep disturbances may be precursors to poor psychological outcomes, and conversely, psychological distress or neurodevelopmental disorders may predict and provoke the development of sleep-related problems.

This Special Issue will explore how sleep quality mainly influences children’s psychological well-being and behavioral profile.

From the evidence collected in this Special Issue, it appears appropriate to suggest that every clinical evaluation of pediatric patients should always include a careful analysis of their sleep habits in order to detect the presence of sleep disorders early and to prevent negative neurodevelopmental repercussions.
